# Elevated Klotho Promoter Methylation Is Associated with Severity of Chronic Kidney Disease

**DOI:** 10.1371/journal.pone.0079856

**Published:** 2013-11-05

**Authors:** Jing Chen, Xiaoyan Zhang, Han Zhang, Jing Lin, Chen Zhang, Qing Wu, Xiaoqiang Ding

**Affiliations:** 1 Laboratory of Kidney Disease, Zhongshan Hospital, Shanghai Medical College, Fudan University, Shanghai, China; 2 Department of Nephrology, Zhongshan Hospital, Shanghai Medical College, Fudan University, Shanghai, China; 3 School of Public Health, Fudan University, Shanghai, China; INSERM, France

## Abstract

Klotho (KL) expression is down-regulated in the renal tissues of chronic kidney disease (CKD) animal models and patients with end-stage renal disease. The putative role of KL promoter hypermethylation in the progression of CKD remains unclear. The present study aimed to determine renal and peripheral blood mononuclear cells (PBMC) levels of KL promoter methylation and analyze their relationship with clinical and histological severity in patients with CKD. Using bisulfite pyrosequencing, renal and PBMC levels of KL promoter methylation were quantified in 47 patients with CKD. 47 nephrectomy specimens of patients with renal cell carcinoma and 48 PBMC specimens of healthy volunteers were used as renal tissue and PBMC controls, respectively. Renal expression of KL protein was assayed by immunohistochemistry staining. Receiver operating characteristic (ROC) curve was used to identify the optimal cut-off value of PBMC KL promoter methylation level for renal KL promoter hypermethylation. Higher levels of KL promoter methylation were observed in renal tissue and PBMC in patients with CKD compared with controls (8.79±3.24 vs. 5.17±1.11%, P<0.001; 7.20±2.79 vs. 3.27±0.79%, P<0.001). In these patients, renal KL methylation level correlated inversely with renal KL immunostaining intensity (ρ=-0.794, P<0.001). Estimated glomerular filtration rate correlated inversely with renal and PBMC levels of KL promoter methylation (r=-0.829, P<0.001; r=-0.645, P<0.001), while tubulointerstistial fibrosis score correlated positively (ρ=0.826, P<0.001; ρ=0.755, P<0.001). PBMC KL promoter methylation level correlated positively with renal KL promoter methylation level in patients with CKD (r=0.787, P<0.001). In ROC curve, the area under curve was 0.964 (P<0.001) and the optimal cut-off value was 5.83% with a sensitivity of 93.8% and specificity of 86.7% to predict renal KL promoter hypermethylation. The degree of KL promoter methylation is associated with clinical and histological severity of CKD. PBMC KL promoter methylation level may act as a potential biomarker of renal KL promoter hypermethylation.

## Introduction

Klotho (KL) is an antiaging gene, which encodes a single-pass transmembrane protein that forms a complex with multiple fibroblast growth factor 23 (FGF23) receptors. KL is most abundant in the renal tubules [1]. KL knockout mice (*KL* ^-^/^-^) develop a syndrome resembling patients with chronic kidney disease (CKD), such as shortened lifespan, hyperphosphatemia, and multiple accelerated age-related disorders including diffusive artery calcification [2]. Recent studies have shown that KL could function as a renoprotective factor [3,4]. KL expression is decreased in the renal tissues of CKD animal models and patients with end-stage renal disease [3,5]. KL expression can be inhibited by chronic circulating stress factors, including oxidative stress, inflammation, uremic toxins and disordered metabolic conditions which are common in uremic status [6]. 

Sun et al [7] demonstrated that the two protein-bound uremic toxins, namely indoxyl sulfate (IS) and p-cresyl sulfate (PCS), could accelerate kidney fibrosis by decreasing KL expression in renal tubules of uninephrectomized B-6 mice via CpG hypermethylation of the KL gene. Specific inhibition of deoxyribonucleic acid (DNA) methyltransferase isoform 1 by 5-aza-2’-deoxycytidine could cause demethylation of the KL gene and increase its expression [7]. Thus, inhibition of KL gene expression correlates with gene hypermethylation, suggesting that epigenetic modification of KL gene may be an important pathological mechanism of uremia [7]. However, the putative role of KL promoter hypermethylation in the progression of CKD is still not clear. Meanwhile, it has been reported that peripheral blood mononuclear cells (PBMC) of patients with CKD present a global DNA hypermethylation characteristics [8]. However, the existence of PBMC KL promoter hypermethylation in patients with CKD still remains unclear. In the present study, the renal and PBMC levels of KL promoter methylation were estimated and the relationship of KL promoter methylation levels with clinical and histological severity in patients with CKD was analyzed.

## Materials and Methods

### Subjects

The study was approved by the Clinical Research Ethical Committee of Zhongshan Hospital, Shanghai Medical College, Fudan University. All patients provided written informed consent. The present study enrolled 47 patients with CKD, who were aged between 18 and 75 years old and selected for renal biopsy at the Department of Nephrology, Zhongshan Hospital of Fudan University, Shanghai, China between April and October 2012. Blood pressure of all patients was lower than 140/90mmHg. Patients with autoimmune diseases, vasculitis, diabetes mellitus, liver cirrhosis, heart failure, acute coronary syndrome, stroke, chronic infectious disease, malignant tumor, previous dialysis, organ transplantation, rapid deterioration of renal function in 3 months prior to the study, smoking habit and previous glucocorticoid or immunosuppressant treatment were excluded. Clinical data including serum creatinine and 24-hour urinary protein excretion rate were recorded at the time of renal biopsy. Estimated glomerular filtration rate (eGFR) was calculated using the Modification of Diet in Renal Disease formula (eGFR=175 x standardized serum creatinine^-1154^. x age^-0.203^ x 0.741 [if Asian] x 0.742 [if female]) [9]. Additionally, the normal renal cortex from the nephrectomy specimen of 47 age- and sex-matched patients with renal cell carcinoma (all clear cell type) and PBMC from 48 age- and sex-matched healthy subjects were considered as renal tissue and PBMC controls, respectively. 

### Sample preparation and DNA extraction

Renal biopsy samples were obtained under ultrasound guidance with a 16-gauge needle. All renal tissues were evaluated by standard light microscopy to ensure that specimens were mainly renal cortex and had at least ten glomeruli. The microscopic procedure also confirmed that only the normal renal cortex was used as renal tissue control. Only those biopsies, which provided a sufficient sample for performing both the standard pathologic examination and molecular biology analysis, were considered for this study. After renal biopsy, the specimens were immediately frozen in liquid nitrogen and stored at -80°C until further processing. Venous blood sample (10ml) was collected in ethylenediaminetetraacetic acid tubes at 4°C before extraction. PBMC were isolated by density gradient centrifugation with Ficoll-Paque Plus (GE Healthcare, Piscataway, USA) as briefly described below. Blood sample was mixed with phosphate buffered saline (PBS) and transferred into a tube containing Ficoll-Paque. After 30 min of centrifugation at 2000 rpm at 18°C, the PBMC layer was extracted, washed twice with Hank's Balanced Salt Solution (Invitrogen, Cergy Pontoise, France), and resuspended in PBS/fetal bovine serum (FBS) (PBS with 4% FBS). After isolation, the cell pellets were stored at -80°C until further processing. Renal tissues and whole blood DNA were extracted using QIAamp DNA Mini kit (Qiagen, Valencia, CA) and Qiagen DNA Blood Mini Kit (Qiagen, Hilden, Germany), respectively; and they were stored at -80°C until processing for analysis. 

### DNA methylation analyses by quantitative bisulfite pyrosequencing

**Figure 1:**
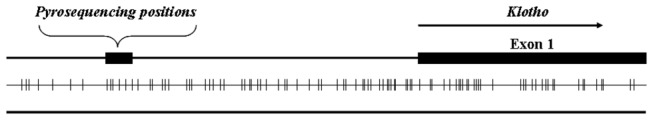
Map of sequencing sites of the Klotho gene. Exon and pyrosequencing positions are shown in top line. CpG dinucleotides are shown in bottomline. Each short vertical bar represents a CpG site. The methylation levels of 6 CpG sites of Klotho are determined.

**Figure 2:**
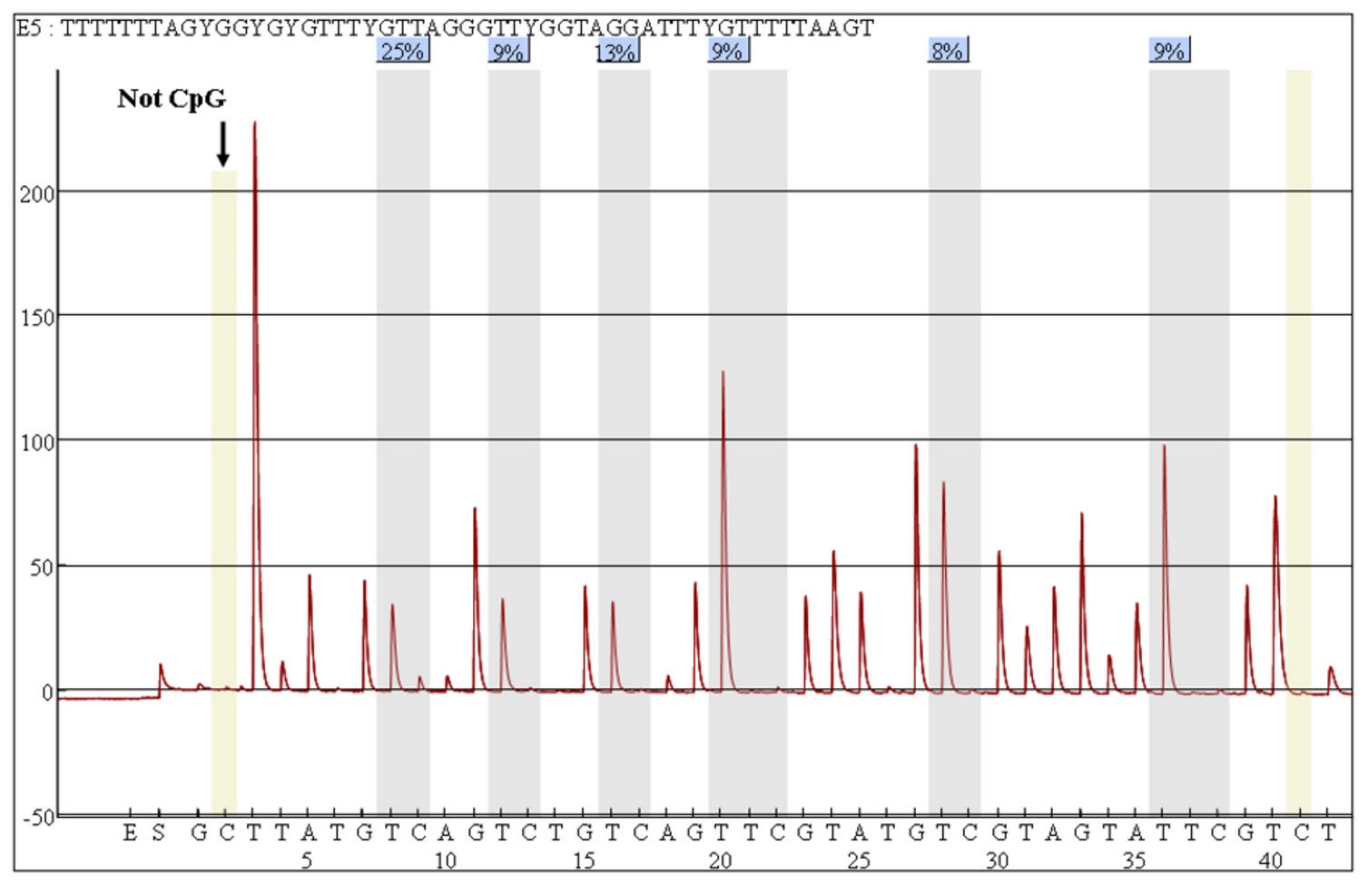
Pyrosequencing assay used to measure Klotho promoter methylation. Methylation level is 12.17% in the renal tissue of a chronic kidney disease patient. The percentage (%) (grey) is the proportion of cytosine at each CpG site after bisulfite conversion, and the methylation level of each CpG site is estimated by the proportion of cytosine (%). The overall Klotho methylation level is calculated as the average of the proportions of cytosine (%) at the 6 CpG sites. Arrows indicate no residual cytosine at the non-CpG site, ensuring complete bisulfite conversion.

DNA methylation analyses were performed on bisulfite-treated DNA using a quantitative assay based on polymerase chain reaction (PCR)-pyrosequencing. Renal tissues and whole blood DNA were treated with sodium bisulfite using EZ DNA Methylation-Gold kit (Zymo Research, CA, USA) per manufacturer's instructions. This process converted all unmethylated cytosine to uracil, which could be recognized as thymidine by Taq polymerase; and it did not affect methylated cytosines. 500 ng of extracted DNA was used for this step. The bisulfite-treated DNA was amplified using Qiagen PyroMark PCR Kit (Qiagen, Hilden, Germany). The locations of CpG sites that were evaluated in KL were identified as described in previous reports [7,10]. The methylation percentage of 6 CpG sites in KL (−432 to −401) was measured (Figure 1) and hypermethylation of these sites had been reported to silence gene expression [7]. The PCR products were amplified using the following primers: F; GTGGGAGAAAAGTGAGAGTAG and R: AAACCCTCAAATTCATTCTCTTTACCTACC-biotinylated. The amplification was carried out as follows: denaturation at 94°C for 15 minutes; followed by 35 cycles each at 94°C for 30 seconds, at 58°C for 30 seconds, at 72°C for 300 seconds; and a final extension at 72°C for 10 minutes. The PCR product was checked by 1.5% agarose gel electrophoresis to confirm the quality and size of the product and rule out the formation of primer dimers. The specific PCR products were then subjected to quantitative pyrosequencing analysis using a PyroMark™ Q96 MD Pyrosequencing System (Qiagen, Germany) per manufacturer's instructions. The pyrosequencing primer for KL was AAGTGAGAG TAGGTG. Non-CpG cytosine residues were used to verify bisulfite conversion. The degree of methylation in the CpG sites tested is expressed herein as the percentage of methylated cytosines (mC) over the sum of methylated and unmethylated cytosine residues. The reported methylation levels are the averages of 6 CpG sites in KL (Figure 2). Each marker was pyrosequenced in two replicates, and the results were averaged. Based on normal samples and internal quality controls, the cut-off value of renal and PBMC KL promoter hypermethylation were set at 7.5% and 5.0%, respectively; and CpG methylation above this limit was considered as hypermethylated.

### Renal KL immunohistochemistry staining

Immunohistochemistry staining for KL was performed using a biotin-streptavidin-peroxidase method as described in a previous report [11]. Rabbit anti-human KL (1:100 dilution; Abcam, Cambridge, UK) and biotinylated goat anti-rabbit immunoglobulin G were used as primary and secondary antibodies, respectively. Sections that were incubated with non-immune rabbit serum instead of the primary antibodies served as negative controls. All sections were stained under identical conditions together with control incubation. Nuclei were counterstained lightly with hematoxylin. The immunoreactivity for KL was scored in a blind manner as follows and was compared with the renal KL methylation levels: 0 for none, 1 for mild, 2 for moderate, and 3 for strong immunostaining.

### Assessment of renal tubulointerstitial fibrosis

Analysis of renal tubulointerstitial fibrosis was determined on 2μm paraffin-embedded sections stained by Periodic Acid-Schiff. The severity of renal tubulointerstitial fibrosis was scored by an experienced pathologist who was blinded to the results of molecular biology studies. For tubulointerstitial lesions, tubular atrophy and interstitial fibrosis were scored as follows: 0 for absent, 1 for mild (involving <25% of the interstitium and tubules), 2 for moderate (involving 25~50% of the interstitium and tubules), and 3 for intense (involving >50% of the interstitium and tubules). A final tubulointerstitial fibrosis (TIF) score was obtained by adding these up and reported between 0 and 6 [12].

### Statistical analysis

All statistical analyses were performed using SPSS (version 17.0, SPSS Inc., Chicago, IL, USA). All the results were presented in mean ± SD for normally distributed data, and median and inter-quartile range for the others. The data of 24-hour proteinuria and eGFR were log transformed due to the large range of values. Chi-square test and t test were used for differences between groups. Correlation was assessed using Pearson and Spearman analysis. Receiver operator characteristic (ROC) curves were constructed to assess the optimal cut-off value of PBMC KL promoter methylation level to predict the renal KL promoter hypermethylation by calculating the true positive rate (sensitivity) against the false-positive rate (1-specificity). Statistical significance was set at P<0.05. All significance tests were two-tailed. 

## Results

### Characteristics of patients with CKD and controls

The demographic and clinical data of the study subjects are summarized in Table 1. As compared to renal tissue and PBMC controls, patients with CKD had significantly higher levels of urinary protein excretion rate and lower eGFR. There was no difference in age and gender between patients with CKD and controls. The underling renal diseases of patients with CKD were IgA nephropathy (n=22), focal segmental glomerulosclerosis (n=8), crescentic nephritis (n=7), minor lesions (n=4), membranous nephropathy (n=4), membranoproliferative glomerulonephritis (n=1), and hypertensive nephropathy (n=1). 

**Table 1:** Demographic and clinical data.

	Patients with CKD (n=47)	Renal tissue control (n=47)	PBMC control (n=48)
Male (%)	51.1	53.1	50.0
Age (year)	42.89±13.91	48.23±9.35	43.84±9.09
Urinary protein excretion rate (g/24h)	1.94 (IQR 0.85-3.48)	0.03(IQR0.00-0.19)*	0.01(IQR0.00-0.10)*
eGFR (ml/min/1.73m^2^)	31.30 (IQR13.59-60.52)	79.70(IQR70.74-101.35)*	101.09(IQR91.55-115.34)*

CKD, chronic kidney disease; eGFR, estimated glomerular filtration rate; PBMC, peripheral blood mononuclear cells.

Compared with CKD patients, * P<0.01.

### Renal and PBMC levels of KL promoter methylation in patients with CKD

**Figure 3:**
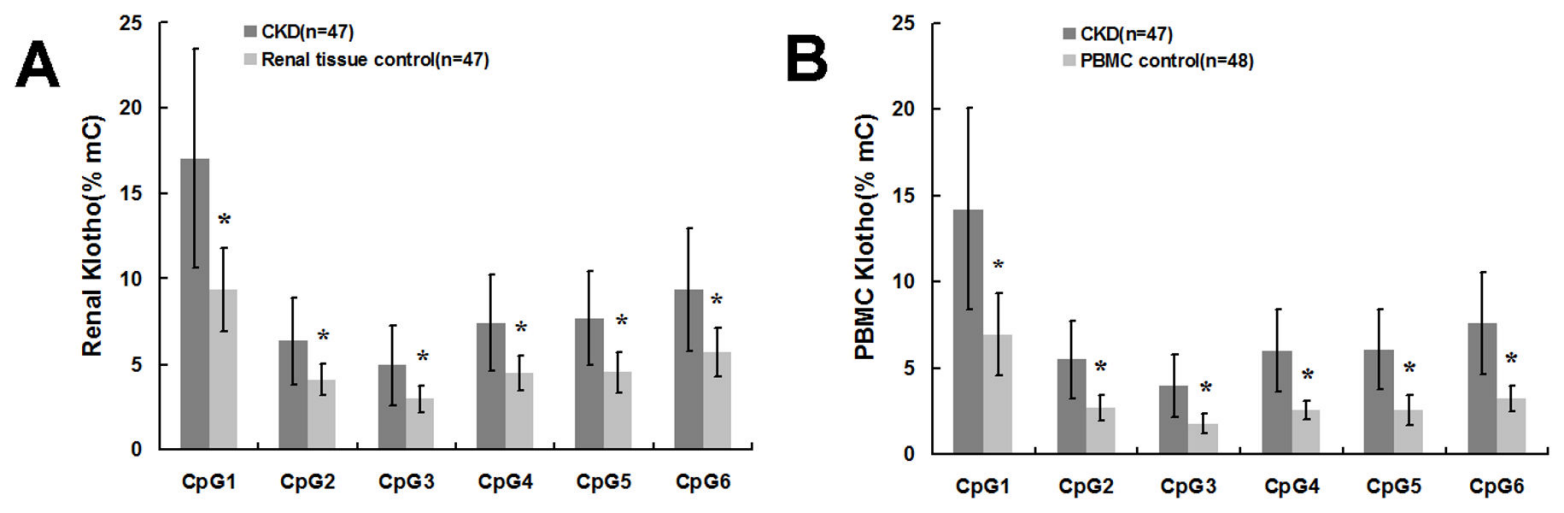
Comparison of methylation levels for 6 CpG islands of Klotho between patients with CKD and controls. A: Renal tissue; B: PBMC. Patients with CKD had higher renal and PBMC methylation levels for 6 CpG islands of Klotho than renal tissue and PBMC control groups. mC%, percentage of methylated cytosines. Compared with the same CpG island of patients with CKD, * P<0.001.

Among 47 patients with CKD, KL promoter was hypermethylated in renal tissue of 32 patients (68.09%) and PBMC of 33 patients (70.21%). KL promoter of renal tissue and PBMC control showed no characteristics of hypermethylation. As compared to renal tissue control, patients with CKD had higher renal level of KL promoter methylation (8.79±3.24 vs. 5.17±1.11%, P<0.001). Similarly, these patients had higher PBMC level of KL promoter methylation than PBMC control (7.20±2.79 vs. 3.27±0.79%, P<0.001). Figure 3 summarized the comparison of methylation level between the patients with CKD, renal tissue control, and PBMC control, which were analyzed for the individual CpG islands of KL. The difference was statistically significant for all 6 CpG sites (p < 0.001).

### Correlation between renal KL methylation level and renal KL immunostaining intensity

**Figure 4:**
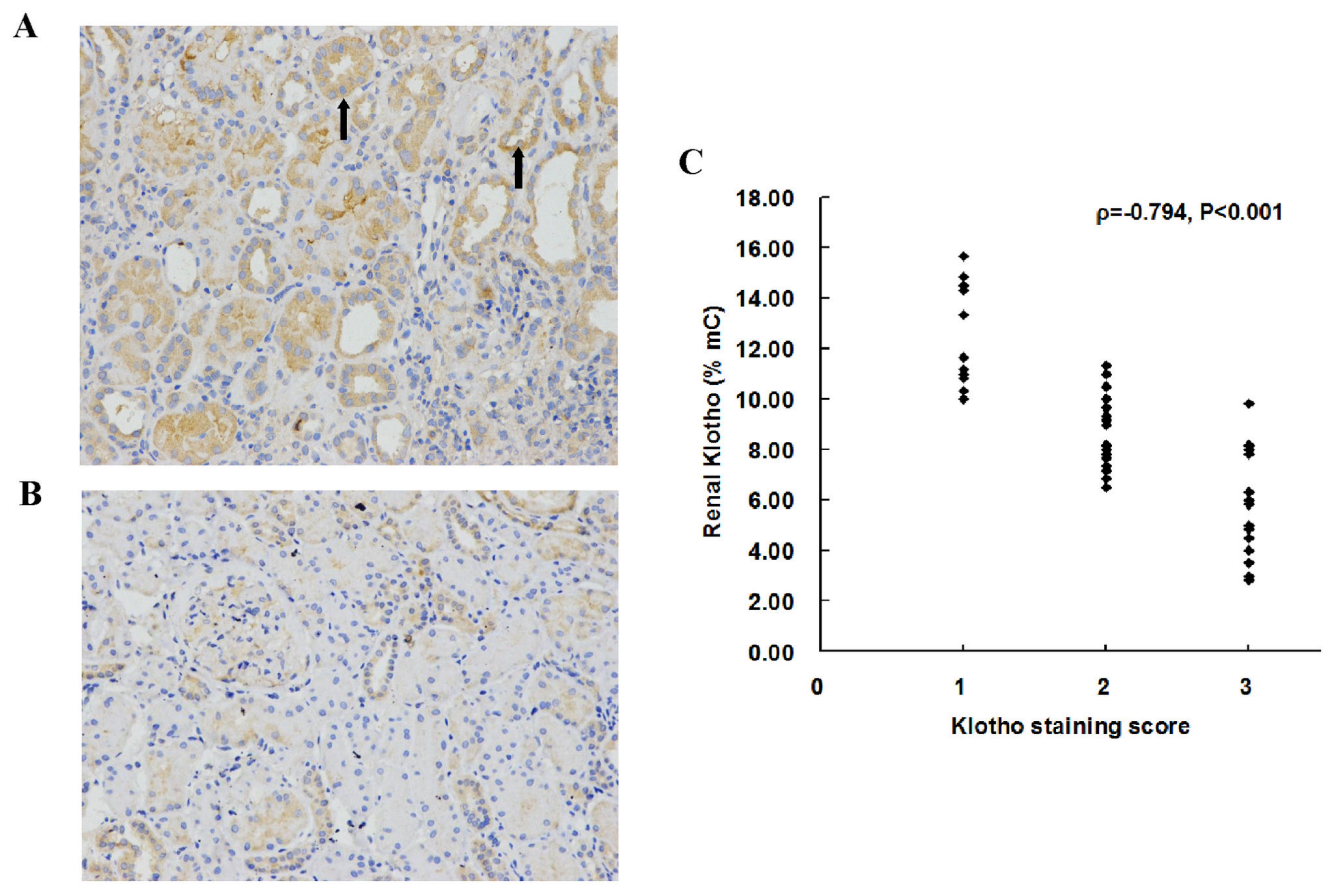
Correlation between renal Klotho methylation level and renal Klotho immunostaining intensity in 47 patients with CKD. Immunostaining of consecutive kidney sections for Klotho in IgA nephropathy (A) and focal segmental glomerulosclerosis (B). Magnification, x200. Arrows indicate the detection of Klotho mainly in renal tubules. The level of renal Klotho methylation correlated inversely with renal Klotho staining score (C). mC%, percentage of methylated cytosines. ρ, Spearman correlation coefficient.

Immunohistochemistry staining of KL was performed using a biotin-streptavidin-peroxidase method to examine correlation between the level of renal KL methylation and renal Klotho protein expression. This was performed in consecutive kidney sections obtained from patients with CKD. Representative pictures of KL immunostaining are shown in Figure 4. KL staining was detected mainly in the renal tubules. Renal KL methylation level correlated inversely with renal KL immunostaining intensity (ρ=-0.794, P<0.001) (Figure 4).

### Correlation between KL promoter methylation levels and clinical or histological parameters in patients with CKD

**Figure 5:**
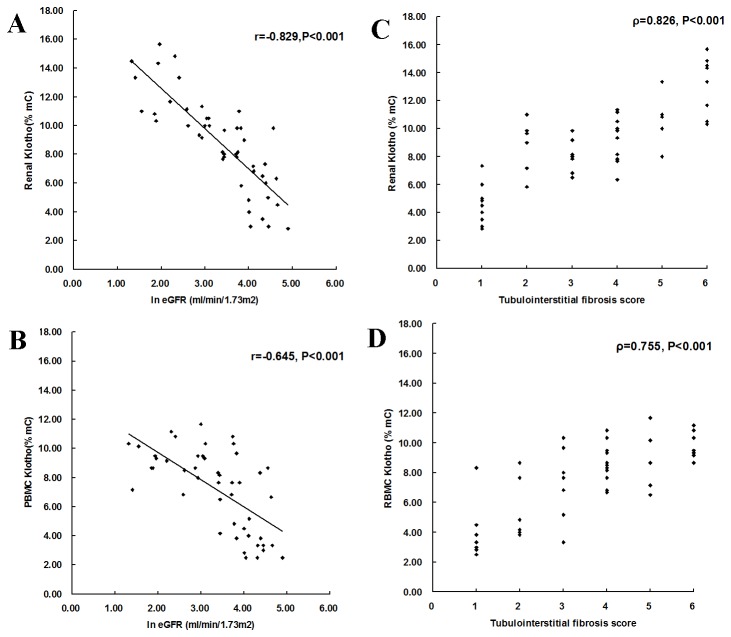
Correlation between Klotho promoter methylation levels and eGFR (A, C) or tubulointerstitial fibrosis (B, D). Renal and PBMC levels of Klotho promoter methylation were found to correlate inversely with eGFR and correlate positively with tubulointerstitial fibrosis score. ln eGFR, log-transformed eGFR. mC%, percentage of methylated cytosines. r, Pearson correlation coefficient; ρ, Spearman correlation coefficient.

In all the 47 patients with CKD, the renal level of KL promoter methylation correlated inversely with eGFR (r=-0.829, P<0.001) and correlated positively with TIF score (ρ=0.826, P<0.001) (Figure 5). PBMC level of KL promoter methylation also correlated inversely with eGFR (r=-0.645, P<0.001) and correlated positively with TIF score (ρ=0.755, P<0.001) (Figure 5). No correlation was demonstrated between 24-hour proteinuria and renal or PBMC level of KL promoter methylation. 

### Correlation between renal and PBMC levels of KL promoter methylation in patients with CKD

**Figure 6:**
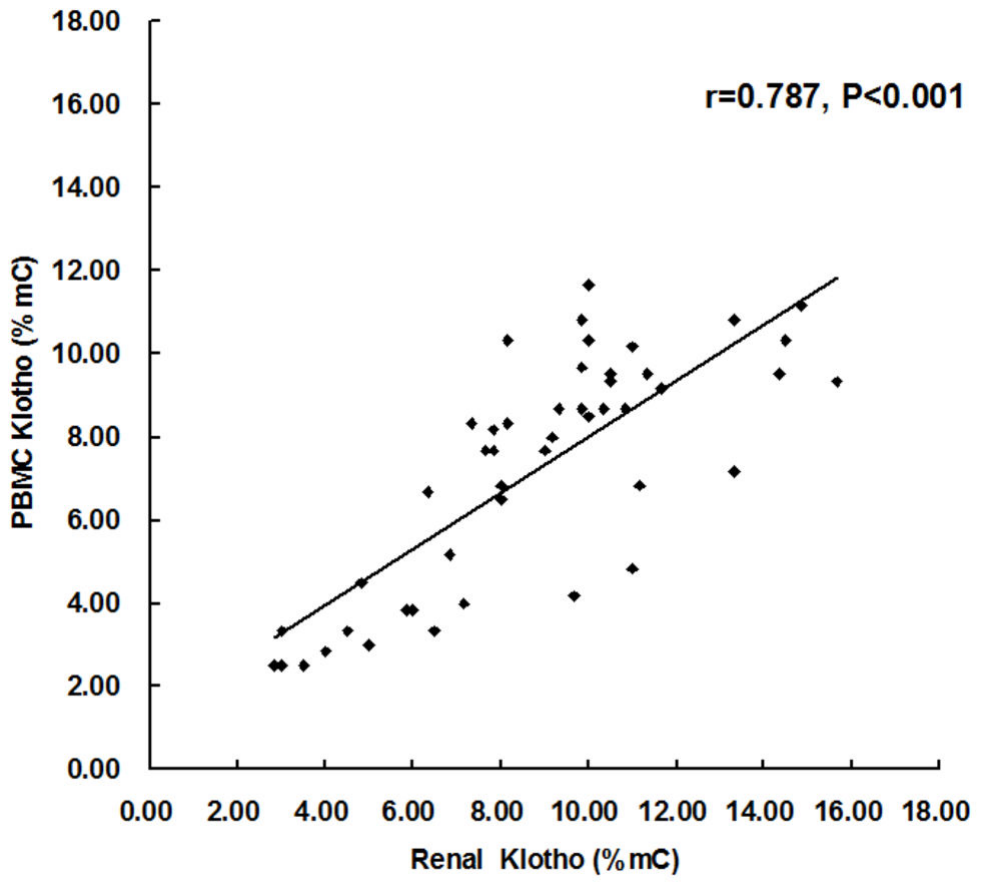
Correlation between renal and PBMC levels of Klotho promoter methylation. PBMC level of Klotho promoter methylation was positively correlated with renal level of Klotho promoter methylation in 47 patients with CKD. mC%, percentage of methylated cytosines. r, Pearson correlation coefficient.

**Figure 7:**
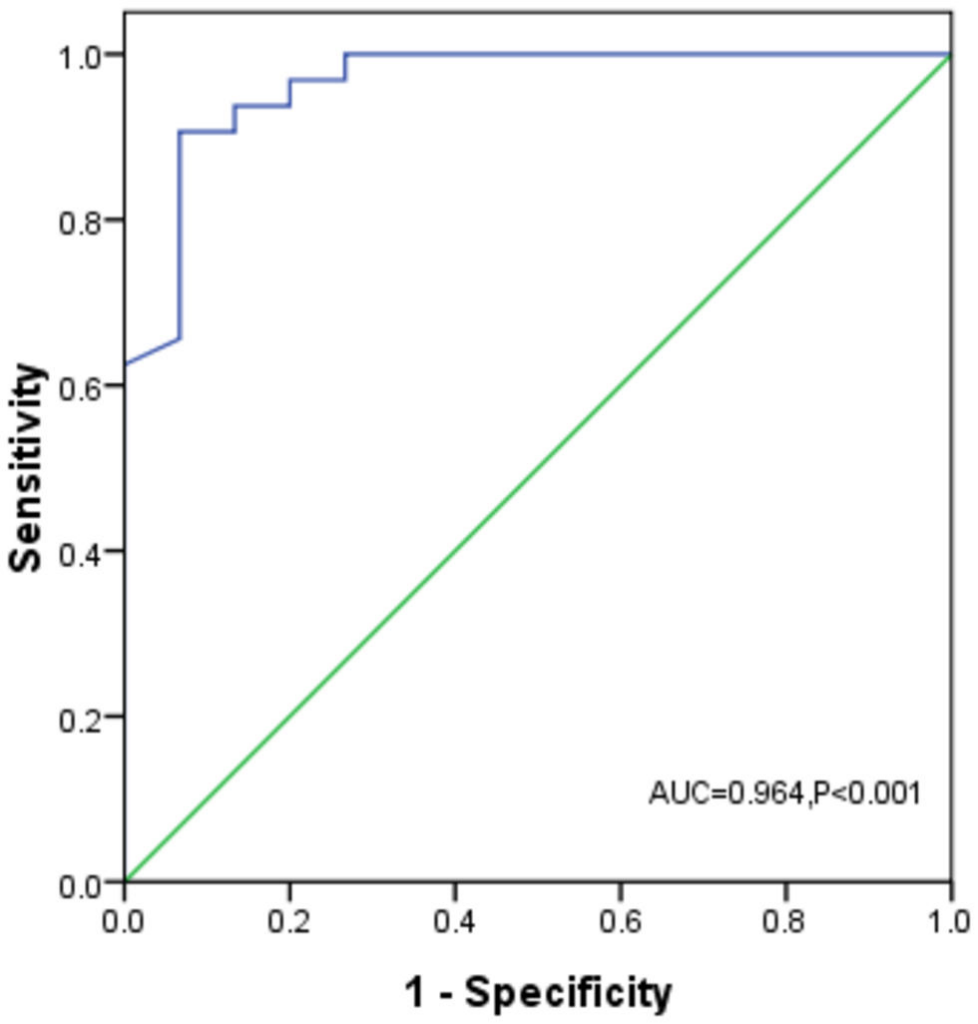
Receiver operator characteristic curve of PBMC level of Klotho promoter methylation to predict renal Klotho promoter hypermethylation. The area under curve was 0.964 (P<0.001) and the optimal cut-off value was 5.83% with a sensitivity of 93.8% and specificity of 86.7% to predict renal Klotho promoter hypermethylation.

PBMC level of KL promoter methylation correlated positively with renal level of KL promoter methylation (r=0.787, P<0.001) in all the 47 patients with CKD (Figure 6). The ability of PBMC KL promoter methylation level to predict renal KL promoter hypermethylation was assessed. ROC curve had a highly significant area under curve (AUC) (0.964, P<0.001). The optimal cut-off value for renal KL promoter hypermethylation showed a PBMC KL promoter methylation level of 5.83%. The best combination of sensitivity and specificity was 93.8% and 86.7%, respectively (Figure 7).

## Discussion

KL is originally identified as an aging suppressor [2]. Klotho is expressed widely, but it is most abundant in the kidney [13,14]. Renal KL messenger ribonucleic acid (mRNA) was found to be lower in a 5/6 nephrectomy model and in nephrectomy samples from patients with end-stage renal disease [5,15,16]. A modest amelioration of proteinuria and renal function were observed when KL was overexpressed genetically in the chronic glomerulonephritis model [3] or via viral delivery in the chronic angiotension ii hypertension model [17]. The same observations were also made in the spontaneous hypertension model [18]. In a recent study, KL was notably decreased in kidney and barely detectable in the blood and urine of CKD model generated using uninephrectomy plus ischemia-reperfusion injury in contralateral kidney [19]. Patients with various stages of CKD had lower levels of KL in their blood and urine. The magnitude of decrease could correlate with the severity of decline in eGFR [19-21]. All these review of literature could suggest that CKD is a state of KL deficiency. The clear mechanism involved in the decrease of KL expression in kidney diseases is presently unknown, but this may potentially include ischemia, oxidative stress, uremic toxins, inflammation, and disordered metabolic conditions [6,13,22-24]. Sun et al [7] demonstrated inhibition of KL gene expression by IS and PCS. Such an inhibition correlated with gene hypermethylation, suggesting that epigenetic modification of KL gene might be an important mechanism for KL deficiency. However, the KL promoter methylation level in patients with CKD remains unknown, and the contribution of KL promoter hypermethylation to the progression of CKD are currently still not clear.

The present study reported the elevation of both renal and PBMC levels of KL promoter methylation in patients with CKD. Renal KL methylation level correlated inversely with renal KL protein expression. The study also demonstrated correlations of renal and PBMC levels of KL promoter methylation with eGFR and histological damage in patients with CKD, suggesting a possible participation of KL promoter hypermethylation in the pathophysiology of CKD. Many mammalian gene promoters are rich in CpG dinucleotide clusters known as CpG islands [25]. Covalent addition of a methyl group to the 5-carbon position of the cytosine is common throughout the genome, but when added to CpG dinucleotides in promoter region, CpG islands generally leads to loss of the associated gene expression [26]. KL promoter hypermethylation can lead to decreased expression of KL protein. The mechanisms by which KL protects kidney from injury include antioxidation, antiapoptosis [23,24], and antisenescence [3]. It was believed that KL functioned mainly, if not uniquely, as a coreceptor for FGF23 binding to FGF receptor 1. FGF23 signal transduction generally requires transmembrane KL as a coreceptor [27,28]. Patients with CKD have a high level of full length FGF23 and upregulation of this signal pathway [29]. It is possible that part of the beneficial effects of KL on kidney in CKD may result from the improvement in FGF23 signal transduction [19]. KL promoter hypermethylation has a potential clinical significance. First, KL promoter hypermethylation can serve as an early and sensitive biomarker of CKD. Secondly, therapy with a demethylating agent may be in the horizon in slowing the progression of CKD.

In a previous study, PBMC gene methylation level was demonstrated as a potential noninvasive biomarker of malignant tumor [30], but no data was available about the PBMC methylation level of specific gene in kidney disease. The present study provided an evidence for the correlation of PBMC levels of KL promoter methylation with eGFR and histological damage in CKD patients. ROC curve of PBMC KL promoter methylation level to predict renal KL promoter hypermethylation had an AUC of 0.964, and the optimal cut-off value was found to be 5.83% with a high sensitivity and specificity. All these data suggested that PBMC KL promoter methylation level might be a potential noninvasive biomarker of renal KL promoter hypermethylation. Progressive decline in renal function is associated with inflammation, augmented oxidative stress, accumulation of diverse toxins, and deranged metabolism, all of which could result in altered epigenetic modifications [31]. Hence, it could be speculated these chronic circulating stress factors were involved in PBMC aberrant DNA methylation in patients with CKD. However, the effect of CKD on PBMC DNA methylation still requires further exploration.

The main limitations of the present study included the following: first, renal cortex tissue was used to detect the renal level of KL promoter methylation without distinguishing glomeruli and renal tubules; secondly, renal KL mRNA expression was unable to be detected due to very scarce samples obtained during renal biopsy and renal KL protein expression was semi-quantitatively assayed by immunohistochemistry staining. It is recommended that future studies should investigate KL promoter methylation level in glomeruli and renal tubules.

## Conclusion

This is the first study to report the pathogenic role of KL promoter hypermethylation in the progression of CKD. This study has also provided evidence that PBMC level of KL promoter methylation closely correlates with renal level of KL promoter methylation, and it may be used as a potential biomarker of renal KL promoter hypermethylation. Further studies are needed to elucidate the utility of KL promoter methylation level as a marker to predict renal fibrosis and to explore the therapeutic efficacy of demethylating agent against the progression of fibrogenesis.
